# Spatial filtering magnetic metasurface for misalignment robustness enhancement in wireless power transfer applications

**DOI:** 10.1038/s41598-023-27719-9

**Published:** 2023-01-11

**Authors:** Valeria Lazzoni, Danilo Brizi, Agostino Monorchio

**Affiliations:** 1 Department of Information Engineering, University of Pisa, 56122 Pisa, Italy; 2 Consorzio Nazionale Interuniversitario per le Telecomunicazioni (CNIT), 43124 Parma, Italy

**Keywords:** Energy science and technology, Engineering

## Abstract

In this paper, we present the design of spatial filtering magnetic metasurfaces to overcome the efficiency decay arising in misaligned resonant inductive Wireless Power Transfer systems. At first, we describe the analytical framework for the control of currents flowing on a finite-size metasurface, avoiding classical truncation effects on the periphery and opportunely manipulating, at the same time, the spatial magnetic field distribution produced by the closely placed RF driving coil. In order to validate the theoretical approach, we conceive a numerical test case consisting of a WPT system operating at 12 MHz. By performing accurate full-wave simulations, we prove that inducing a uniform current in the metasurface results in a more robust WPT system in terms of misalignment with respect to conventional configurations, also including standard metasurfaces. Therefore, while the use of metasurfaces in WPT systems has been already demonstrated to be beneficial in terms of efficiency enhancement, we confirmed that a proper control of the metasurfaces field filtering response can be advantageous also for the misalignment issue. Notably, the free space wavelength at the operating frequency (12 MHz) is 25 m, whereas the proposed metasurface dimensions are only 0.0024λ × 0.0024λ. Despite the extremely reduced dimensions, the spatial magnetic field distribution produced by the closely placed RF driving coil can be nevertheless opportunely manipulated. Finally, experimental measurements conducted on fabricated prototypes validated the numerical results, demonstrating the effectiveness of the proposed approach. These achievements can be particularly helpful in WPT applications where the position of driving and receiving coils frequently changes, as in consumer devices and biomedical implants.

## Introduction

Recently, metamaterials (3D) and metasurfaces (2D) have attracted extraordinary consideration in the electromagnetism community for their remarkable features^1, 2^. As it has been extensively reported in the literature, these artificial materials can be designed with exotic electromagnetic properties, not found in nature, like negative values of dielectric permittivity (ε <  0), magnetic permeability (μ < 0), and negative refractive index (*n *< 0)^3–6^. These effective parameters can be adjusted by specifying the shape and behavior of the resonant unit cells constituting the particular meta-structure under consideration^7–9^. Indeed, metamaterials and metasurfaces are commonly realized through a 3D or 2D array of periodic resonant unit-cells like spiral and split-ring resonators whose dimensions must be in the subwavelength regime. In this way, the electromagnetic waves impinging on metamaterials are myopic to their elementary lattice and they interpret them as homogeneous materials^10–13^. Their potentialities to show desirable electromagnetic properties from radio to optical frequencies have been proved to be extremely useful in several different applications, such as frequency-selective surfaces, electromagnetic absorbers, lenses, and biomedical applications^14–18^.

In particular, magnetic metasurfaces and metamaterials have been also employed in resonant inductive Wireless Power Transfer (WPT). In recent years WPT has become an extremely popular research topic due to the strong interest in removing any type of transmission line from electronic devices^19–26^. Indeed, this technology would allow the transfer of a large amount of power from a driving to a receiving coil, but some limits need to be faced. In particular, the efficiency strongly depends on the coupling coefficient *k* between the driver and receiver coils. To maximize this quantity, the two coils need to be positioned as much aligned as possible such that most of the magnetic flux generated by the driver concatenates through the receiver. Furthermore, a major concern when operating in the near field regime consists in the quick decay of the magnetic field, which is proportional to the cube of the distance. For this reasons, significant effort has been made to design metamaterials and metasurfaces able to enhance the performance of WPT systems. In particular, several works have shown that metamaterials and metasurfaces with negative magnetic permeability can behave as a magnetic flux guide, focusing and enhancing the quickly decaying waves and thereby increasing WPT efficiency^27–31^. In this sense, metasurfaces have different intrinsic advantages over 3D metamaterials, since their reduced thickness is particularly suited for the integration into electronic systems; moreover, they can be easily fabricated, by preserving low costs and reduced ohmic losses^32, 33^. Currently, various types of shapes and configurations of resonating spirals and other printed unit-cells have been studied to demonstrate metasurfaces’ ability to enhance the mutual coupling between magnetic dipoles. Consequently, the inductive link power transfer efficiency, the working distance, and the misalignment robustness of WPT systems can be significantly improved^14, 34–49^. In^42, 46, 47, 50–52^, it was also proved how metasurfaces could be employed to lower E-field peaks, therefore, accomplishing high safety standards for the WPT system while guaranteeing an enhanced power transfer efficiency level with respect to a conventional driver-receiver system.

Typically, the design and analysis of metamaterials and metasurfaces are based on the classical electromagnetic theory where the slab is considered infinite, and uniformly excited by a normal incident plane wave^31^. The overall electromagnetic properties of the infinite slab are retrieved by forcing periodic boundary conditions onto a single unit-cell^53, 54^. However, it is not possible, in practical applications, to fabricate an infinite-size metasurface; in addition, for relatively low-frequency applications like WPT, metasurfaces are expected to be excited by a near-field source. Both these aspects cause undesirable truncation effects so that the metasurface response is not homogeneous as theoretically predicted. Specifically, peripherical unit-cells will respond to the impinging radiation with a lower current than the central ones, leading to a reduction in power transfer efficiency and lower misalignment robustness for WPT systems^55^. In some recent publications, the ability to combine two different types of unit cells within a magnetic metasurface to accomplish field focusing or misalignment robustness in WPT applications has been demonstrated^38, 39^. In addition, another manuscript proposed an eigenmode analysis to modulate the magnetic field distribution by geometrically modifying the structure and, thus, the self-resonance of the elements^52^. Conversely, the works in^56, 57^ presented a WPT system exploiting an optimization algorithm to simultaneously achieve the maximum power transfer efficiency and the optimum trace width and spacing of planar spiral coils that enhance the evenness of the axial magnetic field within a target area above the coil. However, no analytical modeling that can accurately describe and arbitrarily control the metasurface spatial filtering response of the driving coil magnetic field distribution has been introduced.

To overcome these limits, in this paper we propose, for the first time to the best of our knowledge, the design of a passive magnetic metasurface able to opportunely filter the spatial distribution of the driving coil magnetic field and enhance misalignment robustness in Wireless Power Transfer applications by using an analytical approach based on an equivalent circuit model. In particular, achieving a satisfying magnetic field spatial homogeneity is the key factor to overcome the misalignment issue. By making use of the analytical circuit model described in^58, 59^, the response of a magnetic metasurface can be arbitrarily manipulated and controlled, to obtain the desired field distribution. Specifically, in^58^, the authors designed a radio-frequency (RF) solenoid exciting a passive metasurface to validate the analytical model. Nevertheless, no attention has been paid to the potential interactions of the metasurface with an additional RF coil, which is the typical configuration of a WPT system. Therefore, to prove our approach, we start from a typical WPT arrangement consisting of a driving coil (actively fed), a passive receiver, and a metasurface interposed in between these two coils. Firstly, the metasurface is uniformly loaded without the correction for the field homogenization (i.e., affected by truncation effects, otherwise referred to as standard configuration), to verify that it can produce a WPT efficiency enhancement, as already widely demonstrated in the open literature. Hence, we apply the methodology to control the current distribution on the metasurface with the purpose of filtering and homogenizing the driving coil magnetic field distribution to improve the misalignment robustness while preserving the efficiency enhancement of the classical configuration. Finally, the WPT results obtained with the homogenizing metasurface are compared against the simple 2-coil system and the traditionally loaded metasurface.

The remainder of the paper is organized as follows: firstly, a detailed statement of the problem with the specific aim of the manuscript is reported. Then, a section is devoted to present the circuit model and the analytical design guidelines to achieve the metasurface field filtering response control. The WPT set-up and the numerical design for the complete characterization of the metasurface are therefore described. The efficiency and misalignment results from numerical simulations, along with the comparison against experimental results obtained over a fabricated prototype, are discussed. Finally, conclusion follows.

## Results and methods

### Statement of the problem

As reported in the “Introduction”, the purpose of this study is to demonstrate the ability to improve the misalignment robustness of a WPT system by employing a magnetic metasurface able to homogenize the magnetic field distribution produced by a closely placed driving coil.

**Figure 1:**
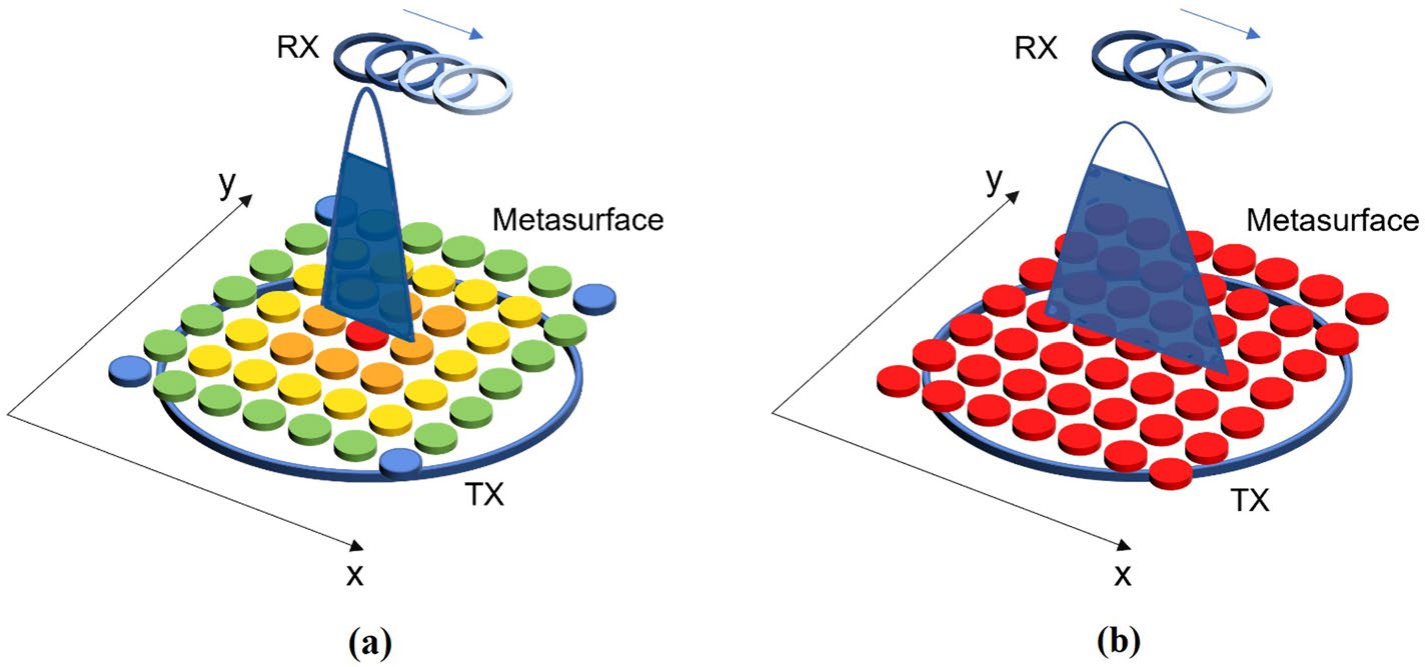
Phenomenology of the WPT systems arrangements presented in this manuscript: (**a**) classical metasurface configuration, suffering from strong truncation effects; (**b**) magnetic metasurface configuration with constant current distribution. In the second case, the magnetic field distribution transverse to the receiver results more homogenous and stronger over a larger area, increasing misalignment robustness.

As it is well known, especially at the low frequency of WPT, metasurfaces are generally constituted by a small number of elements and are excited by a near-field source. Therefore, their actual behavior considerably differs from what is theoretically expected for infinitely extended, plane-wave excited metasurfaces. As shown in Fig. 1a, when a metasurface, for low frequency WPT applications is loaded following the classical theory, the resulting truncation effects play a key role in the system performance. Indeed, the magnetic field distribution (with the blue line is depicted a section of the 3D gaussian profile) is highly focused in a small central area and degrades rapidly towards the periphery. Since the efficiency level is directly related to the magnetic flux linked through the receiver area, system components must be perfectly aligned to achieve the best performance.

This descends from the Maxwell–Faraday law, which explains that the induced voltage at the receiver is proportional to the magnetic field flux into its area:

1 $${{\oint }_{L} \;\underline {e} \cdot \underline {i}_{l} \;dl = - \frac{\partial }{\partial t} {{\iint }}_{S} \underline {b} \cdot \underline {i}_{n} \;dS}$$

To face this issue, we adopted a methodology to analytically control the metasurface filtering behavior in order to homogenize the exciting driving coil magnetic field distribution and minimize truncation effects. By starting from the analyses developed in^58^, a uniform current distribution within the metasurface elements is the ideal configuration for generating a homogeneous magnetic field over a larger area in the near field region. In this way, the obtained magnetic field distribution (Fig. 1b) enables a misalignment robustness enhancement with respect to conventional metasurfaces, while retaining a satisfactory efficiency level. Indeed, the magnetic flux concatenating into the receiver area could be maintained at a higher level even in the presence of a lateral misalignment. The proposed method can be especially useful for all WPT applications in which the exact co-axial alignment between the transmitter and receiver cannot be assured, such as biological implants and consumer electronics.

### Analytical formulation

**Figure 2:**
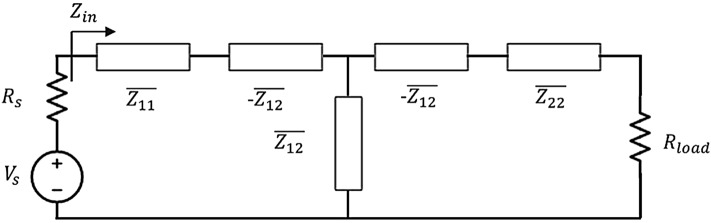
Equivalent 2-port circuit describing the adopted WPT configuration. In the presence of the metasurface, the impedance terms of port 1 and port 2 are modified according to the new values $\overline{{Z_{ij} }}$.

The equivalent circuit model of a generic WPT system with a series load configuration, including a fed driving coil, a passive receiver, and a passive metasurface, is depicted in Fig. 2. Driving and receiving coils and metasurface are planned to operate at the same working frequency *f*_0_. Supposing to identify the driver with the index 1, the receiver with index 2 and the *N* element of the metasurface with the following indices (3, 4, … *N* + 2), the overall system impedance matrix can be expressed as:

2 $$\begin{array}{*{20}l} {\left( {\begin{array}{*{20}l} {Z_{11} } & {\quad Z_{12} } & {\quad Z_{13} } & {\quad \ldots } & {\quad Z_{{1\left( {N + 2} \right)}} } \\ {Z_{21} } & {\quad Z_{22} } & {\quad Z_{23} } & {\quad \ldots } & {\quad Z_{{2\left( {N + 2} \right)}} } \\ \vdots & {\quad \vdots } & {\quad \vdots } & {\quad \ldots } & {\quad \vdots } \\ {Z_{{\left( {N + 1} \right)1}} } & {\quad Z_{{\left( {N + 1} \right)2}} } & {\quad Z_{{\left( {N + 1} \right)3}} } & {\quad \ldots } & {\quad Z_{{\left( {N + 1} \right)\left( {N + 2} \right)}} } \\ {Z_{{\left( {N + 2} \right)1}} } & {\quad Z_{{\left( {N + 2} \right)2}} } & {\quad Z_{{\left( {N + 2} \right)3}} } & {\quad \ldots } & {\quad Z_{{\left( {N + 2} \right)\left( {N + 2} \right)}} } \\ \end{array} } \right)\left( {\begin{array}{*{20}l} {I_{1} } \\ {I_{2} } \\ {c_{3} I_{M} } \\ \vdots \\ {c_{N + 2} I_{M} } \\ \end{array} } \right) = \left( {\begin{array}{*{20}l} {V_{1} } \\ 0 \\ \vdots \\ 0 \\ 0 \\ \end{array} } \right)} \\ \end{array}$$

in which the current flowing in each unit cell of the metasurface has been expressed in the following form:

3 $$I_{i} = c_{i} I_{M} \quad with\quad i = 3,\;4, \ldots ,\;N + 2$$

Thus, *c*_*i*_ is the generic *i*-th complex current coefficient and *I*_*M*_ is an arbitrary reference value^55^.

By adding up equations from row 3 to row *N* + 2 and re-arranging the terms, it is possible to retrieve the equivalent metasurface *RLC* model. Next, the reference current *I*_*M*_ can be expressed as a function of the driver and receiver currents (*I*_1_ and *I*_2_) and the entire WPT system with the interposed passive metasurface can be conveniently described through its equivalent 2-port model:

4 $$\left( {\begin{array}{*{20}l} {\overline{{Z_{11} }} } &\quad {\overline{{Z_{12} }} } \\ {\overline{{Z_{21} }} } &\quad {\overline{{Z_{22} }} } \\ \end{array} } \right)\left( {\begin{array}{*{20}l} {I_{1} } \\ {I_{2} } \\ \end{array} } \right) = \left( {\begin{array}{*{20}l} {V_{1} } \\ 0 \\ \end{array} } \right)$$

where:

5 $$\left( {\begin{array}{*{20}l} {\overline{{Z_{11} }} } &\quad {\overline{{Z_{12} }} } \\ {\overline{{Z_{21} }} } &\quad {\overline{{Z_{22} }} } \\ \end{array} } \right) = \left( {\begin{array}{*{20}l} {Z_{11} - \frac{{Z_{1M} Z_{M1} }}{{Z_{MM} }}} &\quad {Z_{12} - \frac{{Z_{1M} Z_{2M} }}{{Z_{MM} }}} \\ {Z_{21} - \frac{{Z_{M1} Z_{M2} }}{{Z_{MM} }}} &\quad {Z_{22} - \frac{{Z_{2M} Z_{M2} }}{{Z_{MM} }}} \\ \end{array} } \right)$$

and the terms $Z_{MM}$, $Z_{1M}$ and $Z_{2M}$ are the metasurface self-impedance (*RLC* equivalent) and the mutual coupling impedances with the driving and receiving coils, respectively^55^:

6 $$\left\{ \begin{array}{*{20}l} {Z_{MM} = \sum \nolimits_{i = 3}^{N + 2} \sum \nolimits_{j = 3}^{N + 2} c_{j} Z_{ij} } \\ {Z_{1M} = \sum \nolimits_{j = 3}^{N + 2} c_{j} Z_{1j} } \\ {Z_{2M} = \sum \nolimits_{j = 3}^{N + 2} c_{j} Z_{2j} } \\ \end{array} \right.$$

The efficiency of a WPT system, when modelled as a two-port network (4), is usually described as the ratio between the output power dissipated in the useful load *R*_*load*_ and the input power, given as it follows^46^:

7 $$\begin{array}{*{20}l} {\eta = \frac{{P_{out} }}{{P_{in} }} = \frac{{R_{load} \left| {\overline{{Z_{21} }} } \right|^{2} {\mathfrak{R}} \left\{ {\overline{{Z_{in} }} } \right\}}}{{\left| {\overline{{Z_{22} }} + R_{load} } \right|^{2} \left| {\overline{{Z_{in} }} } \right|^{2} }}} \\ \end{array}$$

with:

8 $$\begin{array}{*{20}l} { \overline{{Z_{in} }} = \overline{{Z_{11} }} - \frac{{\overline{{Z_{12} }} \overline{{Z_{21} }} }}{{\left( {\overline{{Z_{22} }} + R_{load} } \right)}}} \\ \end{array}$$

As stated in the “Introduction”, metasurfaces’ design is generally based on rigorous analytical approaches exploiting the classical electromagnetic theory to tailor their properties. The underlined hypotheses to apply such modelization are relative not only to the unit cell size (which must be smaller than the wavelength) but also in considering the metasurface as an infinite array and uniformly excited by an incident plane wave^55^. Nonetheless, the last two conditions are usually far from to be met in a practical scenario, especially at the relatively low frequencies of WPT (order of a few MHz). This means that the metasurface is finite, i.e. made of a limited number of unit cells (few tens), and excited by a driving coil placed in its near-field region. As a consequence, strong truncation effects arise and the performance of the entire system decreases, especially at the border of the selected finite metasurface. Only the central unit-cells respond similarly as theoretically predicted; indeed, they experience an almost ideal infinite condition, since they are surrounded by many other repetitive cells. Instead, the peripheral cells behave differently, according to their specific position within the metasurface.

To face this issue, in this work we employ an analytical framework to arbitrarily control the filtering response of a metasurface excited by a fed driving coil^58^ and coupled with a passive receiver. By imposing the resonant conditions for each array element (from row 3 to row *N* + 2 of (1)) at the chosen working frequency, it is possible to find the reactance that each unit cell must hold to achieve the desired metasurface response:

9 $$\begin{array}{*{20}l} {Z_{ii} = \frac{{ - \sum \nolimits_{j = 3,j \ne i}^{N + 2} c_{j} Z_{ij} }}{{c_{i} }} \quad with\quad i = 3,\;4 \ldots (N + 2)} \\ \end{array}$$

By opportunely selecting the current coefficients *c*_*i*_, any magnetic field distribution can be ideally synthesized by spatially filtering the driver one. In the specific case of this work, we are interested in homogenizing the driver magnetic field distribution, consequently eliminating the truncation effects while preserving the efficiency enhancement produced by classical metasurfaces. As discussed in the previous section, it is required to impose all the current coefficients *c*_*i*_ equal to 1. In this way, the same circulating current can be imposed in each array element and the magnetic field distribution can be homogenized over a larger area. As reported in the next section, this behavior can be used for mitigating the misalignment issue in the WPT application while also accomplishing efficiency enhancement.

### Design procedure

#### Numerical design

**Figure 3:**
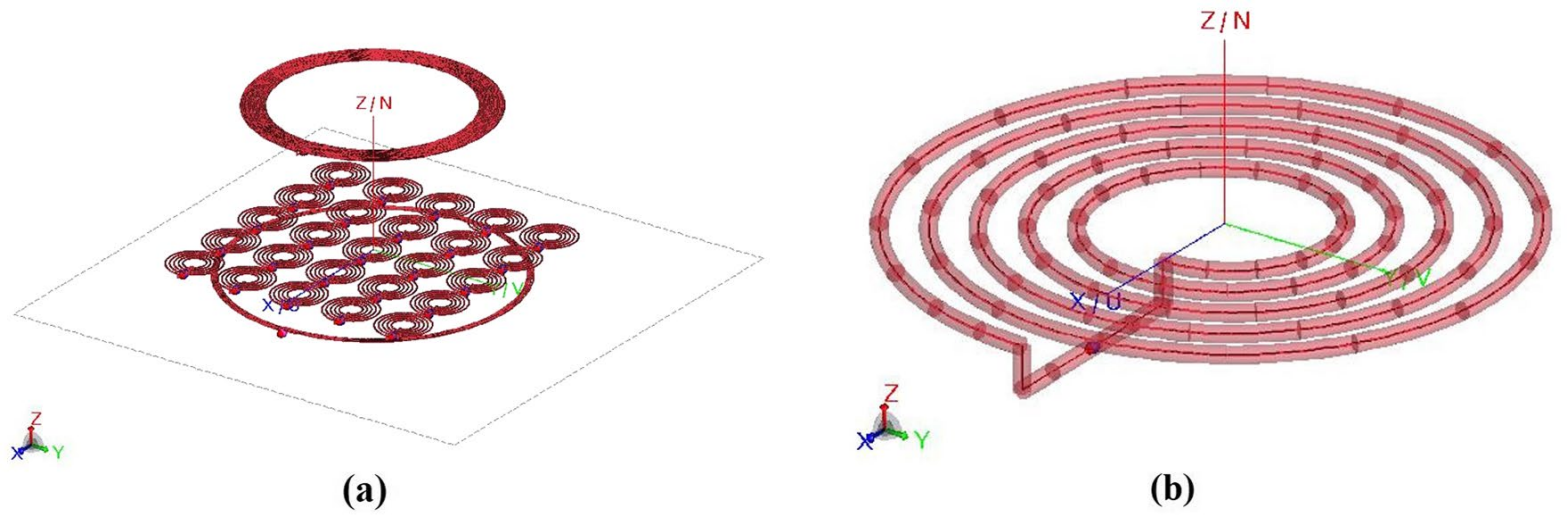
3D CAD model of: (**a**) the WPT system in the presence of the metasurface; (**b**) the single meta-atom constituting the metasurface.

In order to verify the validity of the proposed approach, we designed a typical WPT test case consisting of a fed driving coil, a metasurface, and a receiving passive coil. Fig. 3a shows the CAD model realized using an electromagnetic solver based on the Method of Moments (Feko suite, Altair, Troy, MI, USA).

In detail, we selected an operative frequency of 12 MHz and all the system components were designed by adopting a 28 American Wire Gauge (AWG) lossy copper wire. The driving coil was conceived as a 4-turn spiral coil with an external diameter of 61 mm and a winding pitch of 64 μm. It was made resonant at the desired frequency by inserting a 67 pF capacitor in series with a 50 Ω resistor representing the output impedance of a typical RF power amplifier^46^. Conversely, the receiver was realized as a 14-turn spiral coil coaxially placed 30 mm above the driver; the external diameter, in this case, was 50 mm with a 45 μm pitch. The adequate resonance frequency was ensured by adding a 9 pF capacitor in series with a useful load of 100 Ω. The driving and receiving coils’ parameters are summarized in Table 1.

**Table 1:** Design parameters adopting single strand copper wire.

Parameters	Driving coil	Receiver	Unit cell
Outer diameter (mm)	61	50	11.68
Inner diameter (mm)	58	40	3.48
Number of turns	4	14	5
Copper wire	28 AWG	28 AWG	28 AWG
Added capacitance (pF)	67	9	1030
Source impedance (Ω)	50	N/A	N/A
Series load (Ω)	N/A	100	N/A
Resonant frequency (MHz)	12	12	12

As reported in the “Introduction”, to develop a metasurface for resonant inductive WPT application, it would be theoretically required to create an infinite array of unit cells to fully satisfy the ideal hypothesis and, thus, to avoid undesirable truncation effects. Clearly, such a design is not practically feasible and there are limitations to consider. The metasurface must be as compact as possible with a limited number of unit cells to avoid excessive ohmic losses and to be used in real scenarios. Moreover, it is also important to consider that the metasurface is excited by the driver near-field, which is different from a plane wave excitation. Therefore, we realized a 5 × 5 metasurface, achieving an overall external size of 6 × 6 cm (Fig. 3a); such characteristics are sufficient to enhance the mutual coupling coefficient between the driver and the receiver. In detail, each unit cell (Fig. 3b) was made of a 5-turn spiral resonator with an external diameter of 11.68 mm and a 500 μm pitch, separated by the adjacent one by 1 mm. The overall metasurface was placed 5 mm above the driver and 30 mm below the receiver, in order to maintain the same working distance of the 2-coil system.

At this point, we arranged two different WPT configurations: the “standard” and the “homogenized” case. In the former, each unit cell was made resonant at the same frequency by adding the same capacitor equal to 1030 pF; this is the traditional approach adopted in the literature^14^, i.e. when the array is considered infinite and each cell is surrounded by an infinite number of identical elements. On the other hand, in the second set-up (the “homogenized” case) each cell is loaded with a different capacitor to match the conditions derived from (9) by imposing the same circulating current in each array element (all the current coefficients *c*_*i*_ equal to 1). In this way, the magnetic field distribution produced by the driving coil is homogenized over a larger area. Table 1 summarizes also the unit-cell design parameters for the traditional metasurface approach.

In the following section, the current distributions in the metasurface at the working frequency of 12 MHz and the misalignment robustness for both the arranged configurations will be accurately evaluated. In particular, the energy transfer efficiency for different positions of the receiving coil along the *x*-axis will be used as a metric to assess the validity of the proposed approach to cope with misalignment (Fig. 3a).

#### Prototype fabrication

**Figure 4:**
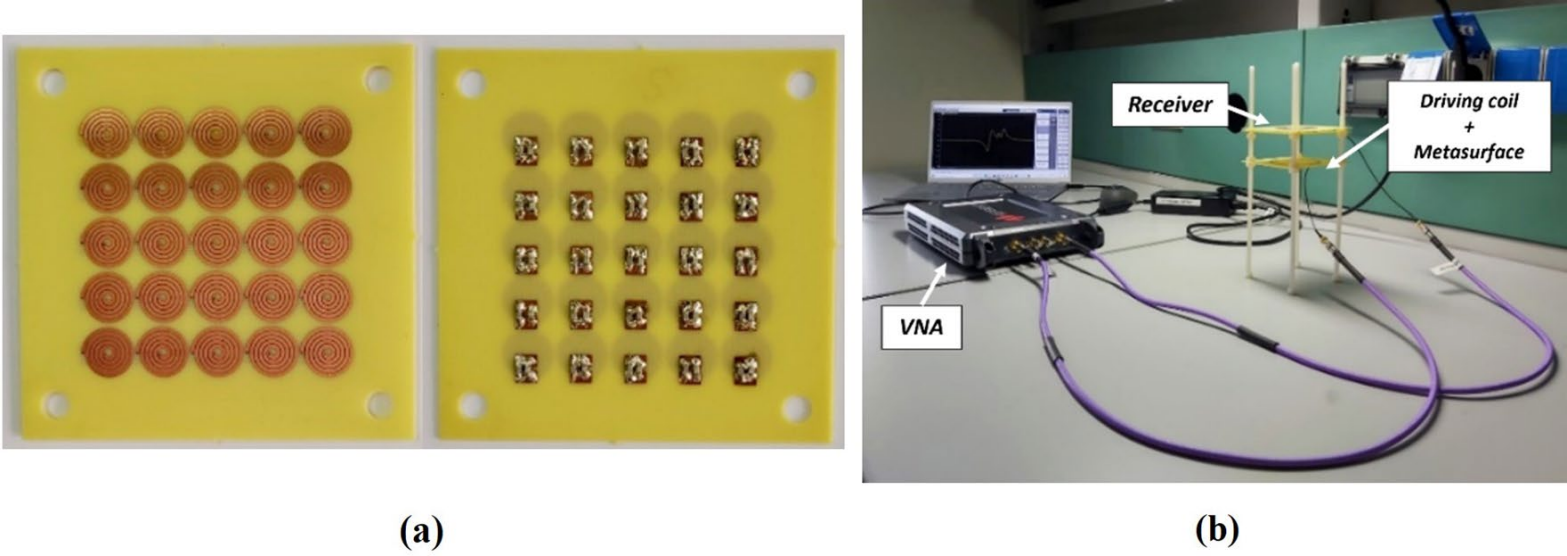
Experimental set-up: (**a**) picture of the PCB metamaterial slab; (**b**) measurement set-up of the WPT system in the presence of the metasurface.

To create an accurate, repeatable, and mechanically robust prototype, all the WPT system components (driver, receiver, and metasurface) have been fabricated by exploiting Printed Circuit Board technology (PCB). We adopted a 0.8 mm thick FR4 substrate over which 35 μm thick copper strips were etched (see Fig. 4a). To minimize the ohmic losses that characterize printed circuit boards^60–62^ and to facilitate the printing process, we converted the geometric parameters of all the three elements (driver, receiver, and metasurface) of the numerically designed WPT system (i.e., with the 28 AWG copper wire) into appropriate strip designs. In Table 2 the experimental system design parameters are summarized. As evident, it was necessary to change the value of the capacitors used to make resonant driver, receiver, and each unit cell of the metasurface (Table 2), due to the different conductor geometry.

**Table 2:** Strip system design parameters adopting PCB copper striplines.

Parameters	Driving coil	Receiver	Unit cell
Outer diameter (mm)	61	50	11.68
Inner diameter (mm)	53	22	1.68
Number of turns	4	14	5
Metal line width (mm)	0.8	0.8	0.8
Spacing between windings (mm)	0.2	0.2	0.2
Conductor thickness (μm)	35	35	35
Added capacitance (pF)	67	9	1030
Source impedance (Ω)	50	N/A	N/A
Series load (Ω)	N/A	100	N/A
Resonant frequency (MHz)	12	12	12

Finally, we soldered surface-mount capacitors over the boards and we connected the driving coil and the receiver to a Vector Network Analyzer (VNA) (VNA P9374A, 300 kHz–20 GHz, Keysight, USA) by using a 50-Ω micro SMA connector. Finally, each substrate slab was provided with four external holes, which allowed to create a nylon support, to guarantee the coaxiality of the structure and the distances between the elements of the system (Fig. 4b).

### Results

#### Numerical results

**Figure 5:**
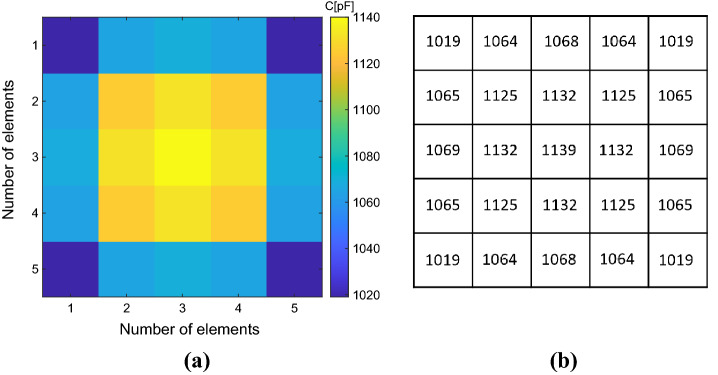
(**a**) Capacitance map calculated through the analytical model previously exposed. The map allows to evaluate the spatial correspondence between each cell of the metasurface and the capacity value required to homogenize its response. (**b**) Capacitance numerical values (pF) used to homogenize the metasurface.

As previously explained, the capacitive loads required to homogenize the driving coil magnetic field, retrieved by the following Eq. (8), are different for each unit-cell of the metasurface. This happens since we imposed the currents flowing in each array element equal to each other by choosing all the current coefficient *c*_*i*_ equal to 1. Therefore, the map of the calculated capacitive loads is depicted in Fig. 5, where their spatial distribution within the array can be also appreciated.

**Figure 6:**
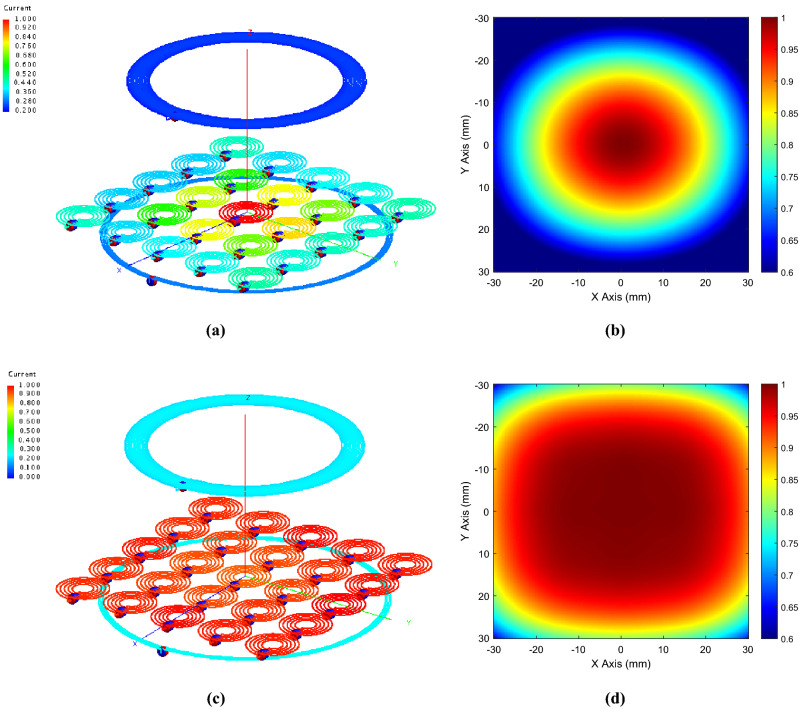
Normalized current distribution within the metasurface elements obtained from a full-wave simulation and corresponding normalized magnetic field at a plane 2 cm away from the metasurface. (**a**,**b**) *Standard case*: since all the unit-cells are loaded with the same capacitors, truncation effects are evident; (**c**,**d**) *Homogenized case*: when the unit-cells are loaded with the proper capacitor to achieve homogenization, the truncation effects are canceled and a more uniform field distribution is obtained.

Afterward, we performed full-wave simulations comparing the two different set-ups: the “standard” and the “homogenized” case. Figure 6 reports the currents and the magnetic field distributions of the two configurations at the working frequency of 12 MHz. Both distributions are normalized to their own maximum values.

**Figure 7:**
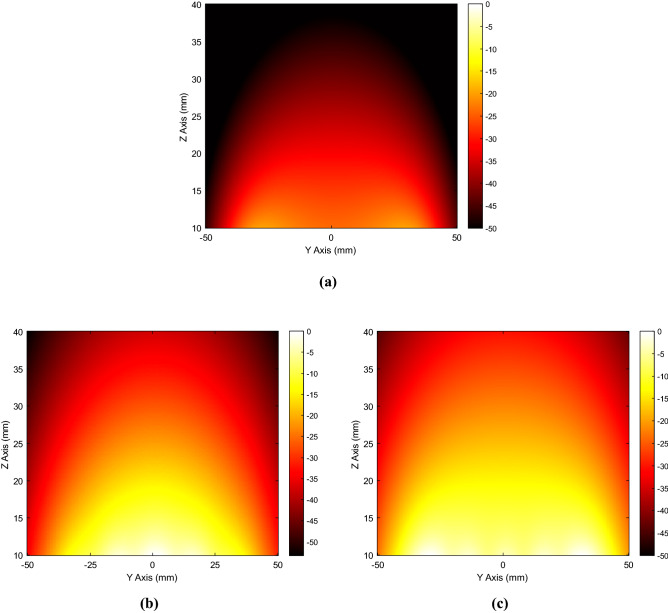
Normalized magnetic field map (normalized to their own maximum values and expressed in dB)) evaluated on a plane perpendicular to the metasurface (*yz* plane in Fig. 3a): fed driving coil without (**a**) and with standard (**b**) and homogenized (**c**) metasurface. To ensure a fair comparison, the same circulating current is forced into the driving coil. As evident, both the metasurfaces are able to enhance the H-field amplitude. However, the unit-cells currents uniformity results in a significantly more homogeneous magnetic field distribution.

As evident, we were able to compensate for the array’s finite size; indeed, the homogenized case does not present any truncation effect and the magnetic field distribution is spread over a larger area with respect to the traditional approach. Furthermore, we also evaluated the H-field maps on a plane perpendicular to the driver (*zy* plane in Fig. 3a), without (driving coil only) and with the presence of the standard and the homogenized metasurface. In order to ensure a fair comparison between the three scenarios, the same circulating current was enforced in the actively fed driving coil. Comparing Fig. 7a and Fig. 7b,c, it is evident that both the metasurfaces configurations are able to enhance the H-field amplitude produced by the driver with respect to the 2-coil system configuration. Nevertheless, as evident, the unit-cells current uniformity leads to a significantly more homogenous magnetic field distribution. As already highlighted, this key feature is fundamental to improve the WPT performance in terms of misalignment robustness. It must be noticed that the spatial filtering behavior of the proposed metasurface can be nonetheless achieved with extremely reduced electrical dimensions. Indeed, while the free space wavelength at the operative frequency of 12 MHz is about 25 m, the metasurface measures only 0.0024λ × 0.0024λ.

**Figure 8:**
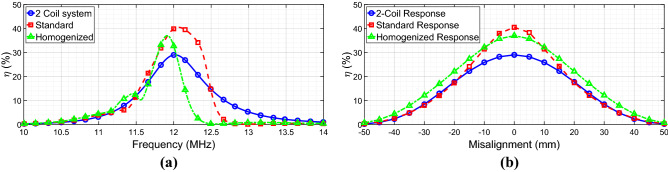
(**a**) Simulated efficiency plots of three configurations: 2-coil system (blue full line), standard metasurface (red dashed line), and homogenizing metasurface (green dashed-dotted line). (**b**) Efficiency versus misalignment: the homogenizing metasurface (green line) can preserve high efficiency values even under misaligned conditions, unlike the standard metasurface (red line) and the 2-coil system (blue line).

After that, we moved to evaluate the power transfer efficiency (see Eq. (6)) of the various configurations at the working frequency of 12 MHz. In particular, we compared the standard case, the homogenized case, and the simple 2-coil system (i.e., constituted by driver and receiver without any interposed metasurface). As expected from the literature, the power transfer efficiency is enhanced in the presence of the standard metasurface compared to the simple 2-coil system (Fig. 8a). Notably, the power transfer efficiency improvement is also achievable with the homogenizing metasurface; therefore, this result underlines the fact that the homogenized configuration increases the mutual coupling between driver and receiver almost in the same manner as in the standard approach, while generating a far more homogeneous field distribution.

Following this observation, we evaluated the misalignment robustness of the three different WPT configurations by calculating the power transfer efficiency for different positions of the receiving coil along the *x*-axis (refer to Fig. 3a for the coordinate system). As it can be deduced from the corresponding results in Fig. 8b, the “homogenized” non-uniformly loaded case is more robust to the misalignment with respect to both the simple 2-coil system and the “standard” uniformly loaded metasurface.

Indeed, for a receiver misalignment greater than 10 mm, the power transfer efficiency achievable with the homogenizing metasurface response is higher due to the more uniform magnetic field and the absence of truncation effects.

#### Experimental results

**Figure 9:**
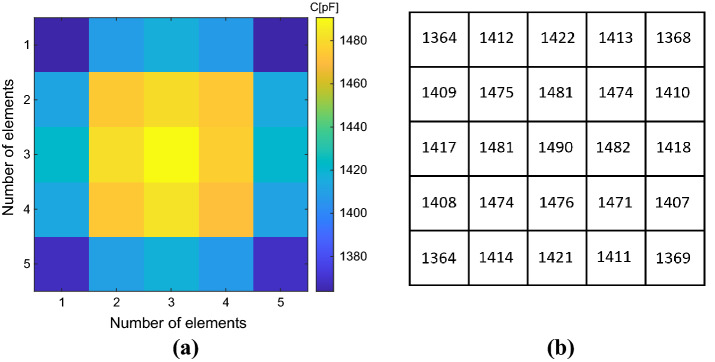
(**a**) Capacitance map required to homogenize the PCB metasurface response calculated through the analytical model previously exposed. (**b**) Capacitance numerical values (pF) used to homogenize the PCB metasurface.

As described in the numerical results section, we also evaluated the capacitive loads required to homogenize the metasurface filtering response according to the analytical model for the fabricated prototype (Fig. 9). Clearly, since the fabricated prototype is made of copper strip lines (and not of a single strand wire), these loading values are different from the numerical example. Nevertheless, from a comparison of Figs. 5 and 9, it can be highlighted that the same spatial behavior can be spotted both for the single-strand wire and the strip line. In this section, we compared the “standard” and the “homogenized” experimental configurations, in terms of efficiency level and misalignment robustness.

**Figure 10:**
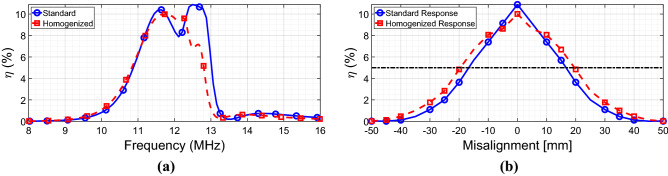
(**a**) Measured efficiency comparison between standard metasurface (blue full line) and homogenized metasurface (red dashed line). (**b**) Measured efficiency versus misalignment: the homogenized metasurface can outperform in terms of misalignment robustness the standard case.

The results in term of efficiency have been reported in Fig. 10a; as a general observation, an efficiency reduction can be observed passing from the numerical to the experimental set-up. This is mainly due to the fabrication process; indeed, PCB traces notoriously suffer from additional ohmic losses with respect to the single-strand wire. Nonetheless, the repeatability and the accuracy of PCB printed boards constitute important advantages, especially for our comparison purposes between the “standard” and the “homogenized” configurations. In any case, the homogenizing metasurface can confer to the WPT system an efficiency level very similar to the standard configuration but with a more robust behavior with respect to the misalignment, confirming the numerical results.

**Figure 11:**
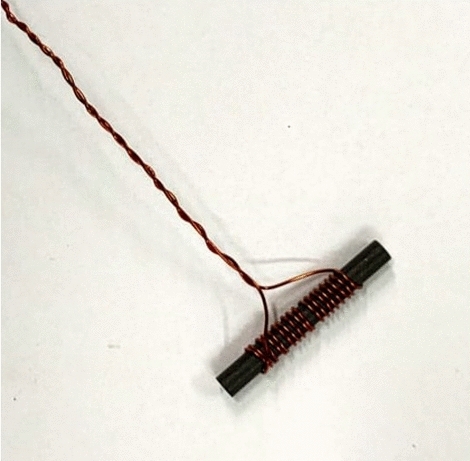
Fabricated uncalibrated magnetic field probe.

In addition, an uncalibrated magnetic field probe has been fabricated in order to assess the current distribution in each cell of the standard and homogenizing metasurface; this aspect is required to experimentally verify if the homogenization filtering procedure reveals effective^63^. The realized sensor is made of a small ferrite cylinder around which a wire solenoid is wrapped (Fig. 11). The sensor is characterized by dimensions small enough compared to the unit-cell so as not to disturb the field distribution (about 25 mm long and with a diameter of 5 mm). We evaluated the voltage drop at the end of the probing solenoid, which is directly proportional to the magnetic field produced by the single unit-cell. In turn, the unit-cell magnetic field is also proportional to the current circulating in it. Therefore, by using a VNA, it has been possible to obtain the circulating current maps at the operating frequency of 12 MHz for both the standard and homogenized configuration. Clearly, since the probe was not calibrated, only normalized current measurements could be obtained.

**Figure 12:**
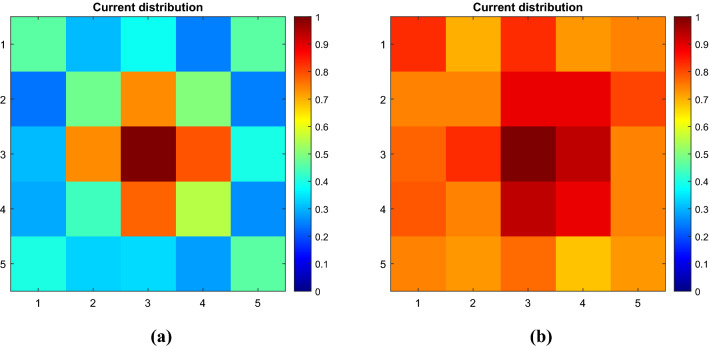
Representation of: (**a**) measured current distribution on the standard metasurface, (**b**) measured current distribution on the homogenized metasurface.

In Fig. 12 the normalized distributions of the measured currents that are flowing in the cells of the standard (uniformly loaded) and homogenized (non-uniformly loaded) metasurface are reported. Both the current distributions are normalized to their own maximum values. As it can be observed, the standard metasurface response is characterized by the same truncation effects observed through full-wave simulations: the maximum current can be found for the central cell and progressively there is a decrease towards the periphery. Conversely, such inhomogeneities are eliminated in the homogenizing metasurface which presents a uniform measured current distribution over the entire surface.

Finally, the misalignment robustness of the two WPT arrangements has been experimentally quantified. As Fig. 10b shows, the homogenizing metasurface is able to confer more robustness to the system; indeed, by starting from a misalignment of ± 10 mm with respect to the *x*-axis, the homogenized configuration is able to outperform the standard metasurface, in good agreement with the full-wave results. In particular, for a fixed efficiency level (5%, black line in Fig. 10b), the WPT system with the homogenizing metasurface is able to handle a total misalignment of 40 mm, compared to a misalignment of 34 mm (+ 17.6%) of the WPT system integrated with the standard metasurface. Clearly, this result is not so marked as obtained in the numerical simulations because the differences among the capacitances required in the peripheral cells are lower than the lumped elements tolerances (± 1%). For this reason, we cannot expect perfect homogenization around the periphery of the metasurface.

## Discussion

In this paper, we proposed the implementation of a spatial filtering magnetic metasurface for enhancing the misalignment robustness in Wireless Power Transfer applications. We first reported the analytical framework required to achieve the desired filtering response control over the metasurface. Then, we demonstrated through accurate full-wave simulations that it is possible to uniform the currents on a finite-size and near-field excited metasurface eliminating all the truncation effects typically present with the classical design approach. In particular, the homogenizing metasurface is not only able to preserve the power transfer efficiency enhancement proper of standard metasurfaces’ configuration, but also to significantly outperform the classical approach in terms of misalignment robustness. In this way, the typical metasurfaces design hypotheses of infinite size and plane wave excitation can be totally overcome. These numerical results were supported by experimental verification carried out on fabricated prototypes, realized through standard PCB techniques.

The proposed solution can be an extremely important advantage in all the WPT applications where the exact co-axial alignment between driver and receiver cannot be guaranteed, as for instance, in biomedical implants and consumer devices.

Further development can be directed to optimize the proposed design for a real-world scenario, for industrial or biomedical applications, taking special care to minimize the ohmic losses inevitably present in the PCB fabrication technique. We also foresee different configurations with active loads that can dynamically change the current distribution, since the theoretical approach here presented can be easily applied to more general cases.

## Data Availability

The datasets used and/or analyzed during the current study are available from the corresponding author on reasonable request.
